# Numerical modelling of the effects of cold atmospheric plasma on mitochondrial redox homeostasis and energy metabolism

**DOI:** 10.1038/s41598-019-53219-w

**Published:** 2019-11-20

**Authors:** Tomoyuki Murakami

**Affiliations:** Seikei University, Department of Systems Design Engineering, Faculty of Science and Technology, 3-3-1 Kichijoji-Kitamachi, Musashino, Tokyo, 180–8633 Japan

**Keywords:** Plasma physics, Computational science, Biochemistry

## Abstract

A biochemical reaction model clarifies for the first time how cold atmospheric plasmas (CAPs) affect mitochondrial redox homeostasis and energy metabolism. Fundamental mitochondrial functions in pyruvic acid oxidation, the tricarboxylic acid (TCA) cycle and oxidative phosphorylation involving the respiratory chain (RC), adenosine triphosphate/adenosine diphosphate (ATP/ADP) synthesis machinery and reactive oxygen species/reactive nitrogen species (ROS/RNS)-mediated mechanisms are numerically simulated. The effects of CAP irradiation are modelled as 1) the influx of hydrogen peroxide (H${}_{2}$O${}_{2}$) to an ROS regulation system and 2) the change in mitochondrial transmembrane potential induced by RNS on membrane permeability. The CAP-induced stress modifies the dynamics of intramitochondrial H${}_{2}$O${}_{2}$ and superoxide anions, i.e., the rhythm and shape of ROS oscillation are disturbed by H${}_{2}$O${}_{2}$ infusion. Furthermore, CAPs control the ROS oscillatory behaviour, nicotinamide adenine dinucleotide redox state and ATP/ADP conversion through the reaction mixture over the RC, the TCA cycle and ROS regulation system. CAPs even induce a homeostatic or irreversible state transition in cell metabolism. The present computational model demonstrates that CAPs crucially affect essential mitochondrial functions, which in turn affect redox signalling, metabolic cooporation and cell fate decision of survival or death.

## Introduction

For aerobic organisms, cellular respiration is one of the most fundamental processes in which oxygen is used to generate energy from carbohydrates (oxidative metabolism). The cellular respiration involving electron transfer is known to be redox, i.e., both reduction and oxidation occurring simultaneously. The redox system involved is essential in maintaining cellular homeostasis through the balance between reactive species generation and elimination, and the redox signalling to and from mitochondria^1,2^. Mitochondria produce significant amounts of reactive oxygen species (ROS) such as hydrogen peroxide (H${}_{2}$O${}_{2}$) and superoxide anions (O${}_{2}^{-}$) during energy metabolism as well as oxidation. Cells can use ROS not only to kill invading pathogens, but also as a key regulator of various signalling pathways^3^. Furthermore, mitochondria have a central role in energy metabolism including adenosine triphosphate (ATP) and adenosine diphosphate (ADP) production and exchange^4^. The integrity of these mitochondrial functions is fundamental to cell life^2,3,5^.

Cold atmospheric plasmas (CAPs) are weakly ionised gases at around atmospheric pressure, in which the electron energy is up to electron volt order while the heavy-particle temperature is around room temperature. Recently, CAPs have been widely applied in the field of biomedicine, such as blood coagulation^6^, inactivation of bacteria, microorganisms or viruses^7,8^, tumour or cancer treatment^9–11^ and induction of apoptotic/necrotic cell death^12,13^. In these works, specific CAP effects on cells and biomolecules via various pathways have been extensively investigated. A number of works have suggested that a strength of CAPs is their ability to deliver multimodal treatments utilising the simultaneous and controllable delivery of electric fields, ions, photons and reactive oxygen/nitrogen species (RONS) both in the gas phase and liquid phase. In particular, the RONS has been implicated as a potential causative agent. ROS such as atomic oxygen, singlet delta oxygen molecules, ozone and hydroxyl radicals as well as H${}_{2}$O${}_{2}$ and O${}_{2}^{-}$ are known as highly reactive species. Some works have pointed out that CAP-induced intracellar ROS generation affects cell survival/death^11,14^. RNS including nitric oxide (NO) and peroxynitrite (ONOO${}^{-}$) in a cell affect mitochondrial transmembrane permeability^15,16^. Recent experimental studies have shown that CAPs deteriorate the mitochondrial transmembrane potential ($\Delta \psi$) and also significantly influence cell survival and death^12,13,17,18^. Table 1 shows the key factors of previous CAP experiments. One of the hypotheses derived from this context is that mitochondrial functions stimulated by CAP-induced RONS generation are critical to biological outcomes. However, a mechanistic understanding of how CAPs exerts their biological effects is still elusive.

**Table 1:** Key factors of some previous CAP experimental works. Potential causative CAP agent, the type of human cell used in experiments and major intracellular effects induced by CAP.

CAP agent	Human cell type	Major intracellular effect	Ref.
H_2_O_2_	PA-TU-8988T, U87MG	H_2_O_2_ change, Apoptotic cell death	^11^
H${}_{2}$O${}_{2}$, OH	3T3-L1	$\Delta \psi$ change, Ca${}^{2+}$ change	^12^
H${}_{2}$O${}_{2}$, NO${}_{2}^{-}$, NO${}_{3}^{-}$	HEK293T	$\Delta \psi$ change, Ca${}^{2+}$ change, Apoptotic cell death	^13^
ROS, RNS	HaCaT	GSH change, Basolateral ATP release	^17^
H${}_{2}$O${}_{2}$, Nitrite	HaCaT, HCT-116, SK-MEL-28	RONS change, $\Delta \psi$ change, Proteasome activity, Oxidative protein damage	^18^

To understand the precise cell functions underlying such effects, systematic biological-reaction models suitable for CAP studies are necessary. To date, various numerical models have been developed to study CAP-induced RONS agents and their multiphase interactions^19^ in the gas phase^20–23^, in the liquid phase^24,25^, at the gas–liquid interface^26,27^ and with biological targets from macroscopic^28–30^ and microscopic viewpoints^31,32^. Despite great progress being made in modelling studies, they have not been applied to intracellular biochemical processes. Significant gaps still exist in our knowledge of fundamental mechanisms that can bridge CAP physics and molecular biology.

**Figure 1:**
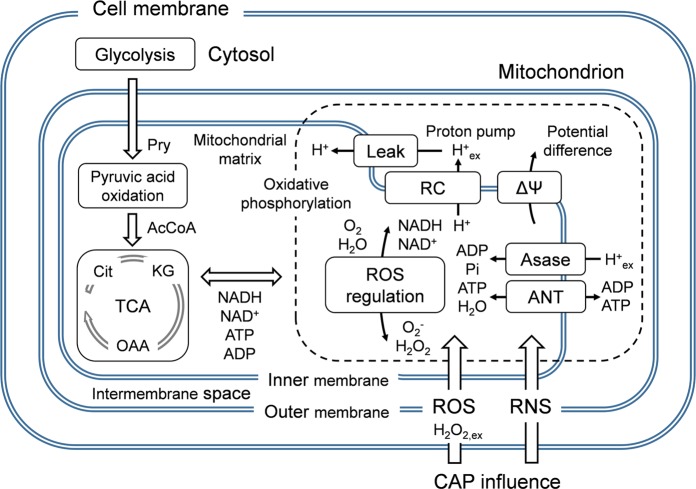
Essential mechanism simulated in the present biochemical reaction model. The effects of externally applied CAP are illustrated. The functions of the cell membrane, cytosol and glycolysis are not taken into account.

The objective of this work is to propose an integrative systematic intracellular-level numerical model to gain new insights into CAP interactions with mitochondrial functions. Figure 1 shows an essential mechanism of mitochondria considered in the present model. The model simulates a mitochondrial redox-mediated function and energy metabolism under the influence of CAP-originating RONS penetration. Specifically, the effects of external H${}_{2}$O${}_{2}$ influx and transmembrane potential disturbance on pyruvic acid oxidation, the tricarboxylic acid (TCA) cycle and oxidative phosphorylation involving the respiratory chain (RC), the ATP synthesis machinery and the ROS-regulation system are examined. This nicotinamide adenine dinucleotide (NAD, i.e., NADH as a reduced NAD and NAD${}^{+}$ as an oxidised NAD)-mediated biochemical network is kinetically solved through a time-dependent zero-dimensional simulation involving 23 biochemical agents and 26 reaction pathways. Two indexes are used to represent CAP-induced changes in parameters: H${}_{2}$O${}_{2}$ inflow flux from the external environment into the mitochondrial matrix ($\Gamma$H${}_{2}$O${}_{2,ex}$ of up to 1.0 $\mu$M/s) and the variation in the transmembrane potential ($\Delta \psi$ of 150 mV $\pm$ 20 mV). The effects of CAP-induced stress on mitochondrial functions are quantified with particular emphasis on the rhythm and stability of ROS, the behaviour of NAD and the ATP/ADP metabolism. The knowledge on how the CAP-induced effects emerge from the whole biochemical dynamics will enable a realistic assessment of the importance of CAP active agents in cellular processes and biomedical outcomes.

## Results

### ROS perturbs redox homeostasis

**Figure 2:**
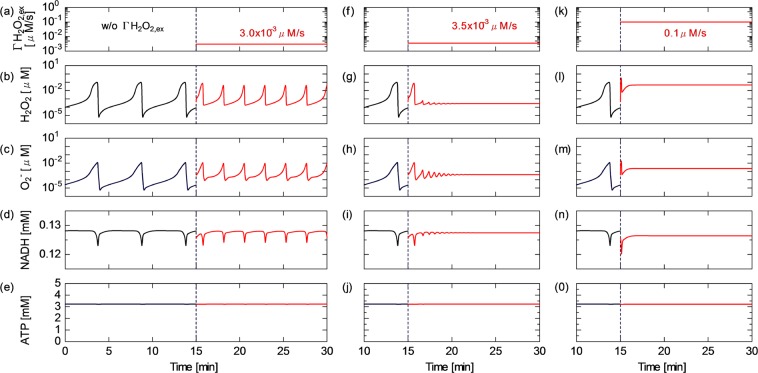
Time courses of the concentrations of intramitochondrial biochemical agents. (**b**,**g**,**l**) H${}_{2}$O${}_{2}$; (**c**,**h**,**m**) O${}_{2}^{-}$; (**d**,**i**,**n**) NADH; and (**e**,**j**,**o**) ATP obtained under the $\Gamma$H${}_{2}$O${}_{2,ex}$ conditions of (**a**–**e**) 0.0 $\mu$M/s at $t$ = 0–15 min and 3.0 $\times$ 10${}^{-3}$ $\mu$M/s at $t$ = 15–30 min, (**f**–**j**) 3.5 $\times$ 10${}^{-3}$ $\mu$M/s at $t$ = 15–30 min and (**k**–**o**) 0.1 $\mu$M/s at $t$ = 15–30 min. All results are obtained at $\Delta \psi$ of 150 mV.

How CAP-induced reactive species perturb redox homeostasis is examined with particular emphasis on the mitochondrial ROS regulation activity. To understand the dynamics of the ROS regulation system and the influence of the external inflow flux of hydrogen peroxide ($\Gamma$H${}_{2}$O${}_{2,ex}$) on the mitochondrial activity, the time courses of H${}_{2}$O${}_{2}$, O${}_{2}^{-}$, NADH and ATP concentrations are shown in Fig. 2, where $\Delta \psi$ is fixed at 150 mV (which is the standard value for normal cell activity). The results before $t$ =15 min are obtained without the CAP influence. As shown in Fig. 2(a)–(c), the H${}_{2}$O${}_{2}$ and O${}_{2}^{-}$ concentrations widely oscillate in the range of 10${}^{-5}$–0.1 $\mu$M in the first 15 min. They show synchronised oscillatory dynamics, whose cyclic period is about 5 min. NADH slightly fluctuates with a ripple factor of 2% [Fig. 2(d)]. The ripple is in phase with the ROS oscillation. In contrast, the ATP concentration is almost constant at 3.2 mM [Fig. 2(e)]. $\Gamma$H${}_{2}$O${}_{2,ex}$ of 3.0 $\times$ 10${}^{-3}$ $\mu$M/s is continuously provided to the system after $t$ = 15 min. The oscillatory dynamics of H${}_{2}$O${}_{2}$, O${}_{2}^{-}$ and NADH are clearly changed owing to the $\Gamma$H${}_{2}$O${}_{2,ex}$ onset. The cyclic period is shortened by about half and the dynamic range (between the maximum and minimum values) of the oscillating amplitude is reduced. Despite this modulation of the rhythm and shape, the steady oscillating behaviour is maintained, suggesting that the regulating activity still works against $\Gamma$H${}_{2}$O${}_{2,ex}$ perturbation. Figure 2(f)–(j) show the species concentrations at a slightly higher $\Gamma$H${}_{2}$O${}_{2,ex}$ of 3.5 $\times$ 10${}^{-3}$ $\mu$M/s. After starting H${}_{2}$O${}_{2}$ application, the dynamics of ROS and NADH gradually switch from the oscillatory to nonoscillatory (stationary) state within 5–10 min. As a result of the oscillatory–stationary transition, the average concentrations of ROS and NADH slightly decrease. At $\Gamma$H${}_{2}$O${}_{2}$ higher than the critical value of 3.5 $\times$ 10${}^{-3}$ $\mu$M/s, the oscillating behaviour settles to the stationary state immediately after the H${}_{2}$O${}_{2}$ onset. At an excessive high $\Gamma$H${}_{2}$O${}_{2,ex}$ of 0.1 $\mu$M/s, the concentration of ROS particularly H${}_{2}$O${}_{2}$ markedly increases, whereas the NADH concentration decreases, i.e., the NAD redox moves from the NADH to NAD${}^{+}$ side [Fig. 2(k)–(o)]. This phenomenon indicates that the ROS regulation activity is overloaded; as a result, redox homeostasis cannot be maintained any more against the excessive CAP-induced perturbation. On the other hand, the ATP concentration is essentially unchanged with time at all values of $\Gamma$H${}_{2}$O${}_{2,ex}$ [Fig. 2(e,j,o)].

**Figure 3:**
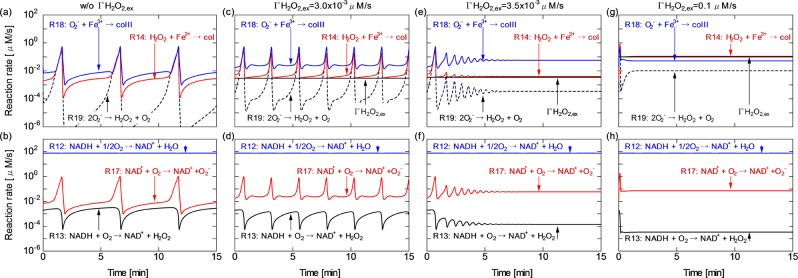
Time series of the reaction rates of the most relevant pathways in the ROS regulation system, (**a**,**b**) without $\Gamma$H${}_{2}$O${}_{2,ex}$. (**c**,**d**) With $\Gamma$H${}_{2}$O${}_{2,ex}$ of 3.0 $\times$ 10${}^{-3}$ $\mu$M/s. (**e**,**f**) With $\Gamma$H${}_{2}$O${}_{2,ex}$ of 3.5 $\times$ 10${}^{-3}$ $\mu$M/s. (**g**,**h**) With $\Gamma$H${}_{2}$O${}_{2,ex}$ of 0.1 $\mu$M/s. $\Delta \psi$ is fixed at 150 mV. The H${}_{2}$O${}_{2}$ onset is at $t$ = 0 s.

Figure 3 shows the time series of the reaction rates of the most relevant pathways involved in the unique dynamics in Fig. 2, from which the reaction scheme among molecular oxygen, peroxidase, enzyme intermediates, NAD (NADH/NAD${}^{+}$), ROS and $\Gamma$H${}_{2}$O${}_{2,ex}$ can be understood. For details of the reaction scheme, see Supplementary Table S1. Figure 3(a) shows the results obtained without $\Gamma$H${}_{2}$O${}_{2,ex}$; the rates of the superoxide dismutase (SOD) reaction (R19) the peroxidase H${}_{2}$O${}_{2}$ detox reaction (R14) and the peroxidase O${}_{2}^{-}$ detox reaction (R18) greatly fluctuate with a cycle period of about 5 min. The reaction rates reach a peak of about 1 $\mu$M/s. H${}_{2}$O${}_{2}$ and O${}_{2}^{-}$ are formed as intermediates during the respiration reaction (R12)^33,34^. It is seen from Fig. 3(b) that the redox transition from NADH to NAD${}^{+}$ is predominantly governed by the respiration reaction (R12), which is constant with time, in contrast to R13 and R17. These minor reactions among the electron donors, H${}_{2}$O${}_{2}$ and O${}_{2}^{-}$ fluctuate similarly to the other oscillating reactions in Fig. 3(a). Figure 3(c,f) indicate that the oscillation of R14 is boosted upward from a low $\Gamma$H${}_{2}$O${}_{2,ex}$. With increasing $\Gamma$H${}_{2}$O${}_{2,ex}$ to 3.0 $\times$ 10${}^{-3}$ and 3.5 $\times$ 10${}^{-3}$ $\mu$M/s, the reaction dynamics markedly change. Not only R14 but also R13, R17, R18 and R19 are influenced by the $\Gamma$H${}_{2}$O${}_{2,ex}$ onset. The dynamic ranges of these oscillations are shrunk and the synchronised cycle period is shortened. Finally, the oscillation is damped to a nonoscillatory state [Fig. 3(e,f)]. As shown in Fig. 3(e), the rate of the H${}_{2}$O${}_{2}$ detox reaction (R14) stops fluctuating in several minutes then remains at 4.0 $\times$ 10${}^{-3}$ $\mu$M/s, which is very close to the $\Gamma$H${}_{2}$O${}_{2,ex}$ of 3.5 $\times$ 10${}^{-3}$ $\mu$M/s. The average rate of R14 decreases to 25% of that obtained without $\Gamma$H${}_{2}$O${}_{2,ex}$. These results suggest that the peroxidase reaction (R14) plays a key role in defence against H${}_{2}$O${}_{2}$. When excessive H${}_{2}$O${}_{2,ex}$ is added, the balance of ROS regulation is lost; all the reactions listed here immediately settle in the stationary state [Fig. 3(g,h)].

**Figure 4:**
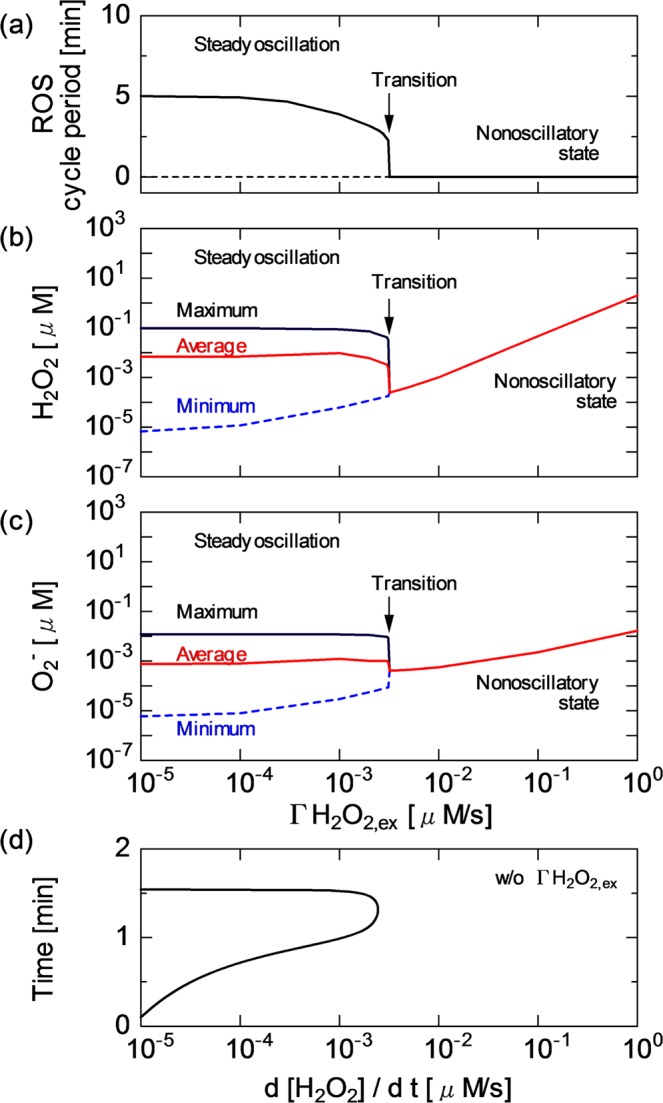
(**a**) Oscillatory cycle period of ROS (H${}_{2}$O${}_{2}$ and O${}_{2}^{-}$) and the concentrations of (**b**) H${}_{2}$O${}_{2}$ and (**c**) O${}_{2}^{-}$ as a function of $\Gamma$H${}_{2}$O${}_{2,ex}$. (**d**) Time differential of H${}_{2}$O${}_{2}$ oscillation in concentration (d[H${}_{2}$O${}_{2}$]/d$t$) without $\Gamma$H${}_{2}$O${}_{2,ex}$. The results are obtained at $\Delta \psi$ of 150 mV.

Figure 4 quantitatively shows the effect of $\Gamma$H${}_{2}$O${}_{2,ex}$ on H${}_{2}$O${}_{2}$ and O${}_{2}^{-}$ stability at $\Delta \psi$ of 150 mV. Both H${}_{2}$O${}_{2}$ and O${}_{2}^{-}$ have the same oscillatory cycle. The period is about 5 min, which is almost constant against the $\Gamma$H${}_{2}$O${}_{2,ex}$ variation below about 10${}^{-4}$ $\mu$M/s [Fig. 4(a)]. Under such low $\Gamma$H${}_{2}$O${}_{2,ex}$ conditions, the dynamic range and average concentration of steady ROS oscillations are essentially unchanged [Fig. 4(b,c)]. The rhythm and shape of ROS oscillation remain constant against the perturbation. This finding indicates that ROS homeostasis is satisfactorily maintained. With increasing $\Gamma$H${}_{2}$O${}_{2,ex}$ to 3.0 $\times$ 10${}^{-3}$ $\mu$M/s, the cycle period gradually decreases from 5 min to approximately 3 min (i.e., the cycle frequency is increasing) and the dynamic range of ROS fluctuation is slightly shrunk (less than 1% reduction). The ROS regulation activity is still efficient at this stage. When $\Gamma$H${}_{2}$O${}_{2,ex}$ rises further and exceeds the critical value of 3.5 $\times$ 10${}^{-3}$ $\mu$M/s (also referred to as the threshold value $\Gamma$H${}_{2}$O${}_{2,exth}$), the ROS oscillates much faster with smaller amplitudes, then fades away with time; the behaviour switches from the oscillatory to stationary state. In this transient phase, the oscillatory and stationary states coexist, i.e., bistability appears in the ROS regulation system. Because ROS stop oscillating [Fig. 4(b,c)], the cycle period cannot be defined (it decreases to zero in Fig. 4(a)). Once the system collapses, it settles in the stationary state at higher $\Gamma$H${}_{2}$O${}_{2}$ values up to 1.0 $\mu$M/s. At this stage, the regulation mechanism cannot defend mitochondria against an excessively high $\Gamma$H${}_{2}$O${}_{2,ex}$. As shown in the high-$\Gamma$H${}_{2}$O${}_{2,ex}$ region in Fig. 4(b,c), the intramitochondrial H${}_{2}$O${}_{2}$ and O${}_{2}^{-}$ concentrations linearly increase with increasing $\Gamma$H${}_{2}$O${}_{2,ex}$. Figure 4(d) shows the time differential of H${}_{2}$O${}_{2}$ oscillation (d[H${}_{2}$O${}_{2}$]/d$t$) obtained without $\Gamma$H${}_{2}$O${}_{2,ex}$. d[H${}_{2}$O${}_{2}$]/d$t$ (positive) corresponds to the inherent intramitochondrial H${}_{2}$O${}_{2}$ production rate under physiological conditions. The peak d[H${}_{2}$O${}_{2}$]/d$t$ is 3.0 $\times$ 10${}^{-3}$ $\mu$M/s, which is very close to the threshold $\Gamma$H${}_{2}$O${}_{2,exth}$ for the state transition. This result clearly indicates that the maintenance of redox regulation is predominantly determined by the balance between $\Gamma$H${}_{2}$O${}_{2,exth}$ and intrinsic intramitochondrial ROS dynamics.

### RNS affects energy metabolism

**Figure 5:**
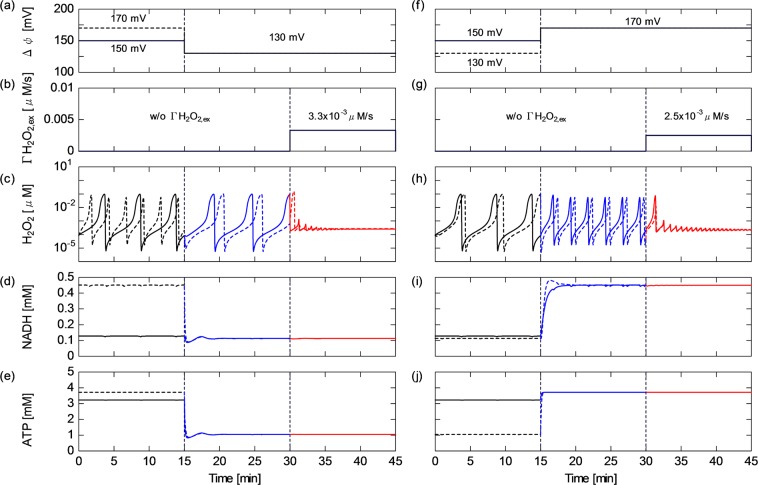
Time series of (**a**) $\Delta \psi$ from 150 mV ($t$ = 0–15 min) to 130 mV ($t$ = 15–45 min) (solid line) and from 170 mV to 130 mV (broken line), (**b**) $\Gamma$H${}_{2}$O${}_{2,ex}$ from 0 ($t$ = 0–30 min) to 3.3 $\times$ 10${}^{-3}$ $\mu$M/s ($t$ = 30–45 min), (**c**) H${}_{2}$O${}_{2}$ concentration, (**d**) NADH concentration and (**e**) ATP concentration. Time series of (**f**) $\Delta \psi$ from 150 mV ($t$ = 0–15 min) to 170 mV ($t$ = 15–45 min) (solid line) and from 130 mV to 170 mV (broken line), (**g**) $\Gamma$H${}_{2}$O${}_{2,ex}$ values from 0 ($t$ = 0–30 min) to 2.5 $\times$ 10${}^{-3}$ $\mu$M/s ($t$ = 30–45 min), (**h**) H${}_{2}$O${}_{2}$ concentration, (**i**) NADH concentration and (**j**) ATP concentration.

The effects of RNS on mitochondrial activity through the RC system is modelled with focus on the $\Delta \psi$ variation, which is directly associated with the adenine nucleotide translocator (ANT) function^16,35^. Figure 5 shows the influences of $\Delta \psi$ and $\Gamma$H${}_{2}$O${}_{2,ex}$ on the energy metabolism and redox regulation. As shown in the period of $t$ = 0–30 min in Fig. 5(a)–(e), the behaviour of ROS as well as that of NADH is little affected by the $\Delta \psi$ reduction from 150 to 130 mV. In contrast, the $\Delta \psi$ reduction markedly decreases the ATP concentration from 3.2 to 1.0 mM (150 $\to$ 130 mV). The $\Gamma$H${}_{2}$O${}_{2,ex}$ onset under the low $\Delta \psi$ condition ($t$ = 30–45 min) switches the H${}_{2}$O${}_{2}$ behaviour from oscillatory to nonoscillatory. As expected from the previous subsection, the O${}_{2}^{-}$ concentration also decreases to the stationary state together with the H${}_{2}$O${}_{2}$ concentration. Figure 5(f)–(j) show that the $\Delta \psi$ increase from 150 to 170 mV after $t$ = 15 min increases the ATP concentration from 3.2 to 3.7 mM. At the same time, the NADH concentration steeply increases fourfold (150$\to$170 mV). Since the change in NADH concentration occurs not only in the TCA cycle but also in the ROS regulation system, it triggers the modulation of the H${}_{2}$O${}_{2}$ dynamics even without the external H${}_{2}$O${}_{2}$ onset. The cyclic periods and fluctuating amplitudes of H${}_{2}$O${}_{2}$ decrease [$t$ = 15–30 min in Fig. 5(c)]. Then, in the period of 30–45 min, the onset of $\Gamma$H${}_{2}$O${}_{2,ex}$ of 2.5 $\times$ 10${}^{-3}$ $\mu$M/s further changes their oscillatory dynamics; strong fluctuation–damping toward the stationary state occurs.

**Figure 6:**
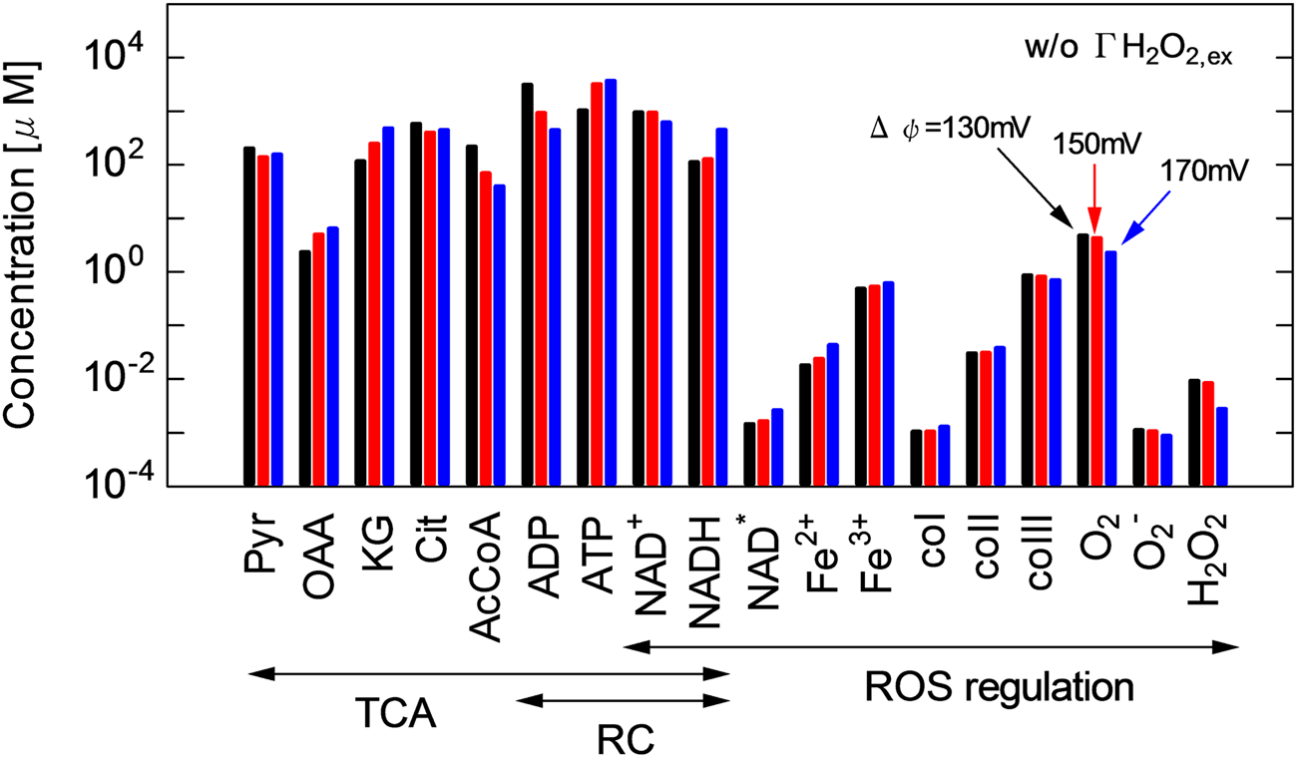
Bar chart of the concentrations of major mitochondrial agents at $\Delta \psi$ of 130, 150 and 170 mV without $\Gamma$H${}_{2}$O${}_{2,ex}$. The mitochondrial agents from Pyr to NADH are involved in the TCA cycle, those from NAD${}^{+}$ to H${}_{2}$O${}_{2}$ in the ROS regulation system and those from ADP to NADH in the RC system.

**Figure 7:**
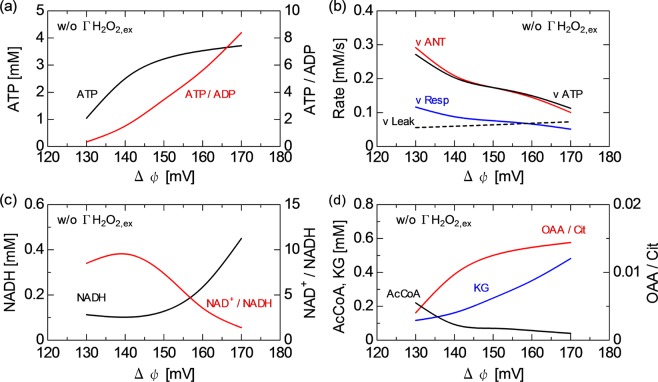
Influences of $\Delta \psi$ on membrane reactions: (**a**) ATP concentration and ATP/ADP ratio and (**b**) rates of major reactions, $\nu$ANT, $\nu$ATP, $\nu$Resp and $\nu$Leak. Influences of $\Delta \psi$ on TCA cycle: (**c**) NADH concentration and NAD${}^{+}$/NADH ratio, and (**d**) AcCoA and KG concentrations and OAA/Cit ratio. These results are obtained without $\Gamma$H${}_{2}$O${}_{2,ex}$. The concentrations and rates are time-averaged for fluctuating values.

To determine the effects of $\Delta \psi$, a bar chart of the average concentrations of major mitochondrial agents at different $\Delta \psi$ is shown in Fig. 6. Pyruvate (Pyr), oxaloacetate (OAA), $\alpha$-ketoglutarate (KG), citrate (Cit), acetyl-CoA (AcCoA), ADP and ATP are involved in the TCA cycle and RC, ferrous peroxidase iron(II) (Fe${}^{2+}$), ferric peroxidase iron(III) (Fe${}^{3+}$), enzyme intermediate compounds I, II and III (coI, coII and coIII, respectively), O${}_{2}$, O${}_{2}^{-}$ and H${}_{2}$O${}_{2}$ are involved in the ROS regulation system, and NAD${}^{+}$ and NADH link these systems. This result is obtained without $\Gamma$H${}_{2}$O${}_{2,ex}$. However, note that the concentration profiles listed here are little influenced by $\Gamma$H${}_{2}$O${}_{2,ex}$ of less than the bistability transient threshold value. Figure 7 shows the influences of $\Delta \psi$ on membrane reactions: (a) ATP concentration and ATP/ADP ratio and (b) the rates of major reactions, $\nu$ANT, $\nu$ATP, $\nu$Resp and $\nu$Leak. It also shows the effects of $\Delta \psi$ on the TCA cycle: (c) NADH concentration and NAD${}^{+}$/NADH ratio and (d) AcCoA and KG concentrations and OAA/Cit ratio. The ATP/ADP ratio is a major indicator of energy metabolic performance. The NAD${}^{+}$/NADH ratio represents the NAD redox activity. The OAA/Cit ratio is an index of the stimulation of NAD-linked dehydrogenases of TCA cycle^36^. For details of the reaction scheme, see Supplementary Table S1.

The key factor to understanding these results is the membrane reaction of RC involving the ATP$\to$ADP exchange, NAD transfer and their association with $\Delta \psi$ variation. Figures 6 and 7(a,b) show that the ATP concentration decreases from 3.7 to 1.0 mM, whereas the ADP concentration increases from 0.4 to 3.1 mM with decreasing $\Delta \psi$ from 170 to 130 mV in the RC system, because $\nu$ANT, which promotes the ATP$\to$ADP exchange (R09), is enhanced from 0.1 to 2.9 mM/s. $\nu$ATP (R11: oxidative phosphorylation to generate ATP) is a competitive pathway for producing ATP and consuming ADP. The rate balance between $\nu$ANT (R09) and $\nu$ATP (R11) predominantly determines the ATP and ADP concentrations [Fig. 7(b)]. Since $\Delta \psi$ reduction corresponds to the degradation of proton pump efficiency, the ADP concentration is enhanced rather than ATP concentration at a low $\Delta \psi$ [Fig. 6]. The increase in the respiration rate ($\nu$Resp, R12) from 0.05 to 0.12 mM/s was induced by stimulating RC [Fig. 7(b)]. Figures 6 and 7(b,c) show that a high $\nu$Resp at a low $\Delta \psi$ favours the NADH$\to$NAD${}^{+}$ transformation; therefore, the NADH concentration is kept low (about 0.1 mM) at 130–145 mV. The NAD transformation tightly links the TCA and RC functions. The NADH concentration steeply increases at $\Delta \psi$ over 150 mV then reaches about 0.45 mM at 170 mV. This reaction is accompanied by the significant consumption of AcCoA; the AcCoA concentration is reduced from 0.22 mM (130 mV) to 0.10 mM (140 mV) and 0.04 mM (170 mV) (see Fig. 7(d)). On the other hand, KG increases with increasing $\Delta \psi$ from 0.12 mM (130 mV) to 0.48 mM (170 mV). OAA also changes similarly to KG. The OAA/Cit ratio declines from 0.013–0.014 at $\Delta \psi$ of 150–170 mV to 0.004 at 130 mV. The mitochondrial membrane potential $\Delta \psi$ is not only critical for maintaining the physiological function of RC to generate ATP but also affects the NAD redox transformation and, therefore, ROS regulation activity. As shown in Fig. 6, the time-averaged concentrations of Fe${}^{2+}$, Fe${}^{3+}$, coI, coII and coIII change with $\Delta \psi$. The time-averaged concentration of H${}_{2}$O${}_{2}$ decreases from 9.3 $\times$ 10${}^{-6}$ to 2.8 $\times$ 10${}^{-6}$ $\mu$M with increasing $\Delta \psi$ from 130 to 170 mV. The O${}_{2}^{-}$ concentration also slightly decreases.

**Figure 8:**
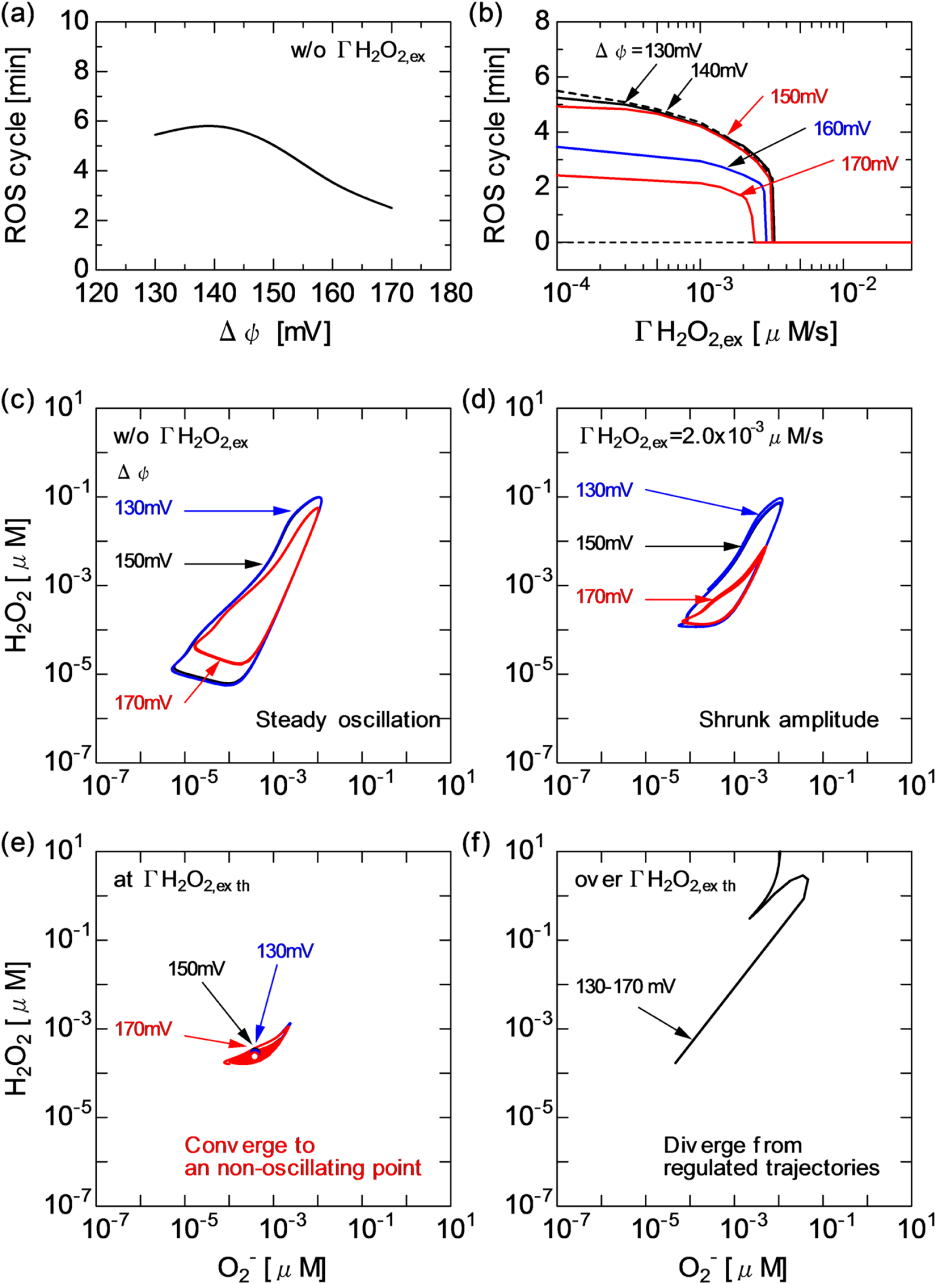
Influences of $\Delta \psi$ and $\Gamma$H${}_{2}$O${}_{2,ex}$ on ROS regulation system, (**a**) oscillatory cycle period of ROS (H${}_{2}$O${}_{2}$ and O${}_{2}^{-}$) as a function of $\Delta \psi$ without $\Gamma$H${}_{2}$O${}_{2,ex}$ and (**b**) ROS cycle as a function of $\Gamma$H${}_{2}$O${}_{2,ex}$ for different $\Delta \psi$ (a merged view is shown to focus on the threshold of transition from oscillatory to stationary state). Trajectories of H${}_{2}$O${}_{2}$ and O${}_{2}^{-}$ concentrations for $\Delta \psi$ of 130, 150 and 170 mV and different $\Gamma$H${}_{2}$O${}_{2,ex}$, (**c**) without $\Gamma$H${}_{2}$O${}_{2,ex}$, (**d**) with $\Gamma$H${}_{2}$O${}_{2,ex}$ at 2.0 $\times$ 10${}^{-3}$ $\mu$M/s, (**c**) at the bistability threshold $\Gamma$H${}_{2}$O${}_{2,exth}$ and (**d**) over the threshold $\Gamma$H${}_{2}$O${}_{2,exth}$.

Figure 8 shows the behaviour of the ROS regulation system at different $\Gamma$H${}_{2}$O${}_{2,ex}$ and $\Delta \psi$. The results allow us to examine the synergetic effects of $\Gamma$H${}_{2}$O${}_{2,ex}$ (caused by ROS) and $\Delta \psi$ (caused by RNS) on the dynamics of mitochondrial activity. As shown in Fig. 8(a), the oscillating cycle period of ROS (H${}_{2}$O${}_{2}$ and O${}_{2}^{-}$) without $\Gamma$H${}_{2}$O${}_{2,ex}$ is kept at 5–6 min for $\Delta \psi$ of 130–150 mV (see also Fig. 5(c)). The TCA cycle and membrane reactions remain essentially the same across the $\Delta \psi$ variation [Figs. 5(d), 6 and 7(c)] at this stage, whereas the cycle period decreases to approximately 2 min for $\Delta \psi$ over 150 mV. This trend is caused by the enhancement of NAD redox (NADH increase and NAD${}^{+}$ reduction) [Fig. 5(i)], which affects the ROS regulation system. Figure 8(b) shows that the threshold $\Gamma$H${}_{2}$O${}_{2,exth}$, at which the transition from the oscillating to nonoscillating state occurs, only slightly decreases from 3.5 $\times$ 10${}^{-3}$ to 2.0 $\times$ 10${}^{-3}$ $\mu$M/s with increasing $\Delta \psi$ from 130 to 170 mV. This observation suggests that the H${}_{2}$O${}_{2}$ onset has a more dominant effect on triggering the transient bistability than $\Delta \psi$.

Figure 8(c)–(f) show the dynamical relationship between H${}_{2}$O${}_{2}$ and O${}_{2}^{-}$ concentrations at different ${\Gamma }_{{H}_{2}{O}_{2},ex}$ and $\Delta \psi$. Trajectories are plotted by collecting the time-evolving concentrations of H${}_{2}$O${}_{2}$ and O${}_{2}^{-}$. The correlation between H${}_{2}$O${}_{2}$ and O${}_{2}^{-}$ concentration dynamics can be understood from the shape of trajectories in the state space. Without the external perturbation of H${}_{2}$O${}_{2}$ concentration, the H${}_{2}$O${}_{2}$–O${}_{2}^{-}$ trajectory steadily rotates along a deformed circle [Fig. 8(c)]. The trajectories at $\Delta \psi$ of 130 and 150 mV are very similar, whereas the area surrounded by the trajectory at $\Delta \psi$ of 170 mV is rather small. With $\Gamma$H${}_{2}$O${}_{2,ex}$ increasing to 2.0 $\times$ 10${}^{-3}$ $\mu$M/s, the surrounded areas are shrunk, particularly at high $\Delta \psi$ [Fig. 8(d)]. As shown in Fig. 8(e), when $\Gamma$H${}_{2}$O${}_{2,ex}$ approaches the bistability threshold and beyond, the trajectories tend to remain in a narrow area and further converge toward a point (i.e., converge to the nonoscillatory state). At a much higher $\Gamma$H${}_{2}$O${}_{2,ex}$, the trajectory leaves its original orbit with a marked increase in the concentration of H${}_{2}$O${}_{2}$ independent of $\Delta \psi$ [Fig. 8(f)].

## Discussion

The first half of this paper focused on the intramitochondrial H${}_{2}$O${}_{2}$ dynamics and emphasised how the rhythm was modified by CAP-originating H${}_{2}$O${}_{2}$. The range of extracellular and intracellular ROS concentrations and the related transport phenomenon across biomembranes (diffusion) have been extensively investigated^37–42^. Most works have shown that the extracellular H${}_{2}$O${}_{2}$ concentration was in the range of 10${}^{-1}$–100 $\mu$M^37–39^ under homeostatic physiological conditions. The intracellular physiological H${}_{2}$O${}_{2}$ concentrations have been measured to be between 10${}^{-3}$ and 10${}^{-1}$ $\mu$M^38^. The gradient of the H${}_{2}$O${}_{2}$ concentration between the extracellular and intracellular (cytosol) components has been estimated to be about 7-fold^40^, 20-fold^39^, 100-fold^38^ or 650-fold^41^. It has also been reported that externally provided H${}_{2}$O${}_{2}$ permeates rapidly across biomembranes^42^. This ROS diffusion is controlled by mitochondrial permeability transition pores (MPTPs) and inner membrane anion channels as well as $\Delta \psi$ variation. The gradient across the mitochondrial membrane has been assumed to be smaller than the gradient between the extracellular and intracellular components^40^. Thus, the present numerical results showing that the intramitochondrial H${}_{2}$O${}_{2}$ concentration fluctuates in the range of 10${}^{-5}$–10${}^{-1}$ $\mu$M (10${}^{-2}$ $\mu$M on average; Figs. 2, 4(b), 5(c,h), 6 and 8(c)) are plausible taking into consideration the 100 $\mu$M concentration for extracellular H${}_{2}$O${}_{2}$, the 100-fold gradient for cellular membranes and the 10-fold gradient for mitochondrial membranes.

As shown in Fig. 2, the concentrations of H${}_{2}$O${}_{2}$ and O${}_{2}^{-}$ oscillate with a cycle period of several min to maintain the inherent redox balance without CAP-induced perturbations. Biochemical oscillations occur in metabolic networks as a result of various modes of cellular regulation^43^. The oscillatory dynamics of ROS is considered to be essential when ROS function as secondary messengers in cell signalling processes The characteristic oscillating cycle period numerically simulated here is approximately 5 min or less [Figs. 2, 3, 4(a), 5 and 8(a,b)], which quantitatively agrees with the experimentally reported intracellular oscillatory periods of half a minute to several minutes^34,44,45^.

The present simulation clarified that the H${}_{2}$O${}_{2}$ influx disturbed the redox homeostasis by changing the ROS oscillatory rhythm [Figs. 2, 4, 5 and 8]. With the greater effects of $\Gamma$H${}_{2}$O${}_{2}$, the oscillatory cycle period was shortened; ultimately, the mitochondrial ROS defence system collapsed owing to excessive H${}_{2}$O${}_{2}$-induced stress. The ROS regulation system cannot overcome the disturbance completely, resulting in ROS oscillation being considerably modified, then fading out to settle in the stationary state. It has been experimentally shown that metabolic oscillations involving peroxidase–oxidase reactions could be affected by external chemical disturbances^45^. The transient bistability is often found in biological systems^33,43^. The model revealed that a key factor of the transient bistability was the balance between $\Gamma$H${}_{2}$O${}_{2,ex}$ and intrinsic H${}_{2}$O${}_{2}$ dynamics and quantified that the critical $\Gamma$H${}_{2}$O${}_{2,ex}$ was comparable to the maximum intramitochondrial H${}_{2}$O${}_{2}$ formation rate (the positive peak value of d[H${}_{2}$O${}_{2}$]/d$t$), that is, on the order of 10${}^{-3}$ $\mu$M/s [Fig. 4]. This value quantitatively agrees with the experimentally obtained H${}_{2}$O${}_{2}$ production rate of 10${}^{-3}$ $\mu$M/s^37^.

The above-mentioned effects occur directly as a result of interactions between CAP-induced ROS generation and cells. In the second half of this paper, the change in transmembrane permeability induced by RNS is modelled as a secondary response to other CAP-induced stimuli. Furthermore, synergetic effects of ROS and RNS provided simultaneously are examined. Nitric oxide radicals rapidly react with O${}_{2}^{-}$ to produce an extremely strong and reactive oxidizing agent, ONOO${}^{-}$. RNS, particularly ONOO${}^{-}$, freely penetrates mitochondrial membranes^46^ and acts on ANT in the permeability transition pore complex of MPTPs. The ANT promotes the ATP$\to$ADP exchange across the inner membrane accompanied by a concomitant change in $\Delta \psi$^15,16,35^. Excessive inhibition of mitochondrial respiration by RNS results in the opening of MPTPs, which leads to the degradation of $\Delta \psi$. To avoid complexity in modelling the mechanism of RNS interactions with MPTPs, the present calculation employed the formula for ANT reactions and used $\Delta \psi$ as a numerical parameter^35^ without quantitative arguments on intramitochondrial RNS concentration.

**Figure 9:**
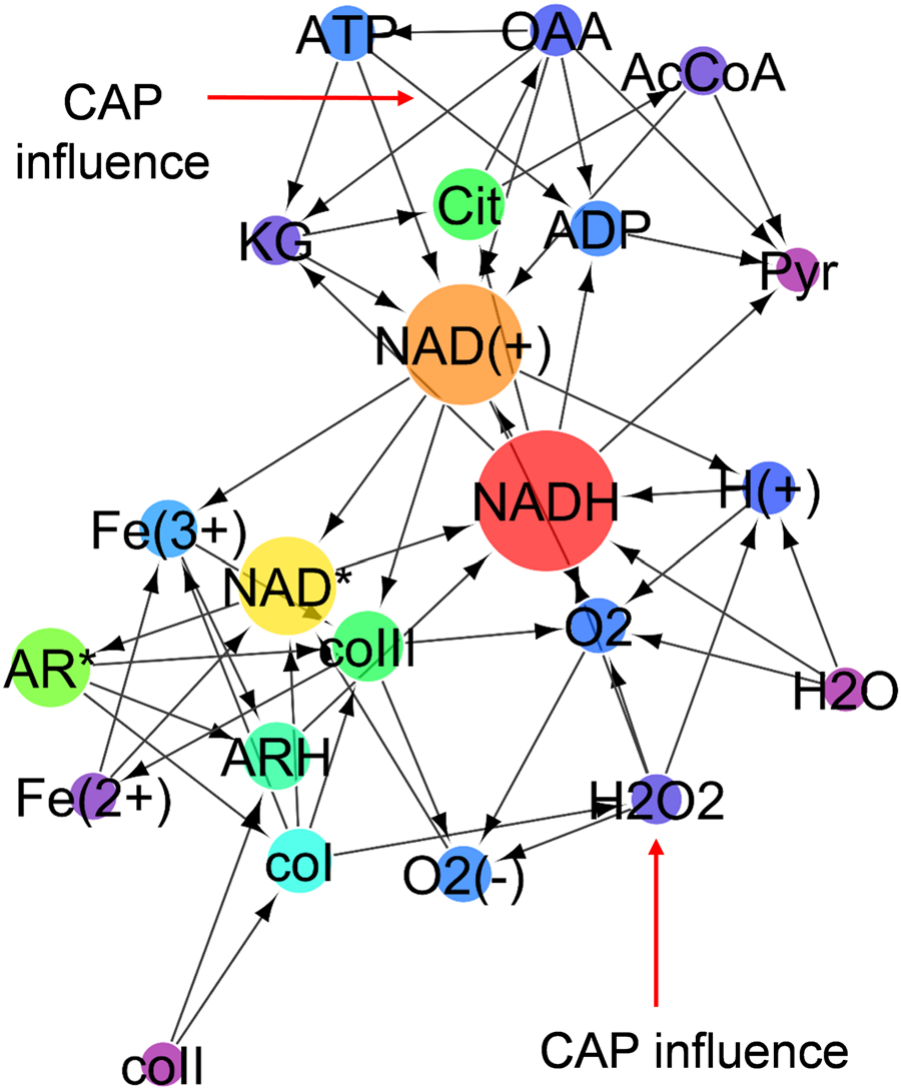
Directed graph of the present biochemical reaction network. Nodes represent species in chemical reactions with its betweenness centrality value as the circle size. Edges start from sources and end at the products of each reaction. The CAP influences are indicated as external edges.

Mitochondrial respiration and ATP synthesis are not only regulated via the linked pathways of energy metabolism but also directly controlled by the NAD${}^{+}$/NADH ratio and oxygen concentration^36^. As indicated by the reactions in Fig. 3(b,d,f,h), NAD is closely linked with ROS and intermediates of the peroxidase reaction system^33,34,37,45^. Figure 9 shows the topology of the present biochemical network, to which graph theory is applied to analyse the network centrality^47^. A directed graph consists of nodes representing species in reactions with its betweenness centrality as the circle size and edges starting from sources and ending at products of each reaction. The betweenness centrality is defined as the ratio of the number of shortest directed paths passing through a given node to that of all the directed shortest paths. The betweenness centrality represents the importance of roles as intermediates bridging between sources and products directly or indirectly. The species are categorised roughly in two groups; agents mainly functioning the TCA cycle and the membrane (upper area of Fig. 9) and agents in the ROS regulation system (lower area of Fig. 9). NADH and NAD${}^{+}$ are located between them and take high betweenness centrality values (their node sizes are largest), indicating that they are the most influential and consolidate the entire network. It is confirmed by network analysis that the model topology is consistent with the fact that the cellular redox state, especially the NAD redox state, is a primary control site in numerous biological processes.

As predicted using the network topology [Fig. 9], the numerical results showed that $\Delta \psi$ is affected by the membrane RC reaction, the TAC cycle and the ROS regution system [Figs. 5, 6, 7 and 8]. Furthermore, as discussed already, the system behaviour is significantly influenced by $\Gamma$H${}_{2}$O${}_{2,ex}$. The key agent bridging them is NAD. Figures 5, 6, 7 and 8 show how the concentrations and dynamics of agents and relevant reaction probabilities changed with $\Delta \psi$ as well as with $\Gamma$H${}_{2}$O${}_{2,ex}$. $\Delta \psi$ has been assessed for many years. Most works have estimated that $\Delta \psi$ is typically in the range of 100–200 mV under physiological conditions^5,36,48^. The present $\Delta \psi$ in the range of 130–170 mV is within this physiological range. Figures 5, 6 and 7 indicate that a low $\Delta \psi$ decreases the ATP concentration and ATP/ADP ratio, resulting in a significant deterioration of energy metabolism. In contrast, a high ATP concentration and a high ATP/ADP ratio at $\Delta \psi$ of over 150 mV would lead to the release of ATP from the cell through ion channels. Figure 7(c) shows that the decrease in $\Delta \psi$ promoted NADH oxidation, i.e., the NADH concentration declined and the NAD${}^{+}$/NADH ratio increased. It has been experimentally shown that an increase in the NAD${}^{+}$/NADH ratio from 0.01 to 10 decreased ROS production activity under the NADH concentration of approximately 0.5 mM^49^. The NAD${}^{+}$/NADH ratio and NADH concentration in Fig. 7(c) are in agreement with the reported values. Figure 7(d) implies that the increased TCA cycle activity lowers the AcCoA concentration, which is also consistent with previous observations^36^.

It has been experimentally demonstrated that CAP irradiation induced the change in mitochondrial transmembrane permeability^12,17^. Some works have shown that CAPs decreased $\Delta \psi$ and increased the intracellular Ca${}^{2+}$ level^12^ and others suggested that CAP irradiation induced stress-dependent ATP release^17^. These CAP-induced intracellular reactions seem to result either in cell survival or death. The ROS dynamics shown in Fig. 8 may provide an explanation for the cell fate decisions. The ROS oscillatory dynamics was changed with $\Gamma$H${}_{2}$O${}_{2,ex}$ and $\Delta \psi$ owing to the activation of NADH-supported ROS formation via the RC system [Fig. 8(a,b)]. With the greater effects of $\Gamma$H${}_{2}$O${}_{2}$ and $\Delta \psi$, the plot of the ROS passage changed from simple periodic oscillation to damped oscillation [Fig. 8(c,d)]. In a cell, the natural rhythm of NAD redox and ROS regulation was impaired. When a negatively regulated state (inherent homeostasis) turned into an irreversible state, overall cell functions were disrupted [Fig. 8(e,f)]. The CAP-originating RONS could synergetically manipulate critical cell functions and affect cell fate decisions.

## Conclusion

The biochemical modelling and numerical simulation quantitatively clarified for the first time how cold atmospheric plasmas affect cell fate decisions by controlling the mitochondrial redox homeostasis and energy metabolism. The CAP-originating ROS and RNS synergetically affected the activities in the TCA cycle and oxidative phosphorylation involving the RC, the ATP synthesis machinery and the ROS regulation system in mitochondria. In particular, RONS stimuli crucially affected the behaviour of ROS oscillation, NAD redox and ATP to ADP conversion. One of the important quantitative findings was that the key factor for the change from cellular homeostasis to irreversibility was the balance between the CAP-induced H${}_{2}$O${}_{2}$ influx and the intrinsic intramitochondrial H${}_{2}$O${}_{2}$ dynamics. Furthermore, it was clearly demonstrated that the RNS-driven change in transmembrane permeability controlled the redox regulation system and ATP/ADP metabolism through NAD-mediated reactions. Such an interplay of CAP-originating RONS with mitochondrial functions may affect not only a single cell function but also further cell–cell interactions. The proposed computational approach is expected to make a strong contribution to the fundamental understanding of the biophysics of CAP–cell interactions by unravelling potentially synergetic effects between multiple agents, which may affect intracellular dynamics in other contexts. The CAP-induced mechanism described in this paper is likely to be a widely applicable principle on how cells achieve homeostasis or lose control of their functions against externally imposed disturbances.

## Computational Methods

A time-dependent zero-dimensional model for mitochondrial functions is developed on the basis of previously published works^33–35,37,44,45,48^. The biochemical reaction set treated here mainly consists of pyruvic acid oxidation, the TCA cycle, membrane reactions and peroxidase–oxidase reactions as a cellular ROS regulation function. The model is essentially built up through a combination of two different models developed in previous works^33,35^. 26 elementary reactions among 26 biochemical agents are considered. The reaction pathways and the related rate constants are shown in Supplementary Table S1. The list of agents, their abbreviations and initial and/or boundary conditions are shown in Supplementary Table S2. This is a simplified model. For instance, although the mitochondrial respiratory chain includes transmembrane complexes I–IV in reality, these functions are assembled in the rate $\nu$Resp *f*([NADH], $\Delta \psi$) for reaction R12 in this model^35^. An expansion of reaction set to examine membrane-bounded complexes and an increase in agent number involving some common biochemical species, e.g. Ca${}^{2+}$, K${}^{+}$, Flavin mononucleotide, Flavin adenine dinucleotide, ubiquinone or cytochrome are the most important aspects of future work. BioModels Database (https://www.ebi.ac.uk/biomodels/) is used to develop the computational codes. Numerical simulations are conducted using Systems Biology Workbench-compliant supporting Systems Biology Markup Language format (http://sbml.org/) together with CellDesigner (http://www.celldesigner.org/). The rate equation governing the reaction kinetics can be expressed as $d{n}_{i}/dt={n}_{i}{\sum }_{j}{k}_{ij}{n}_{j}+{n}_{i}{\sum }_{j}{\sum }_{m}{k}_{ijm}{n}_{j}{n}_{m}+{R}_{ex}$, where ${n}_{i}$, ${k}_{ij}$, ${k}_{ijm}$ and ${R}_{ex}$ denote the concentration of the $i$th species, the two-body reaction rate for the $i$ and $j$th species, the three-body reaction rate for the $i$, $j$ and $m$th species and the external rate source term, respectively. The numerical code solves the differential equation system using Gillespie’s direct method, which is a stochastic simulation algorithm^50^. For details of the numerical scheme, see the literature^33,35^. The CAP influences (the $\Gamma$H${}_{2}$O${}_{2,ex}$ infusion and $\Delta \psi$ change) are added to steady-state or periodic-state solutions obtained for a time series of 10000 s for given initial conditions. The range of the $\Gamma$H${}_{2}$O${}_{2,ex}$ infusion is 0–1.0 $\mu$M/s (constant in time). $\Delta \psi$ (constant in time) is set in the range of 130 mV (de-polarization) – 150 mV (steady state polarization) – 170 mV (hyper-polarization). The time step of 1.0 s is sufficiently shorter than the characteristic time of macroscopic behaviour of mitochondrial agents on which the present model focuses.

## Supplementary information

Supplementary Material
