# Supplementary material for: Numerical modelling of the effects of cold atmospheric plasma on mitochondrial redox homeostasis and energy metabolism
Source: Sci Rep. 2019 Nov 20;9:17138. doi: 10.1038/s41598-019-53219-w (PMC6868247; doi:10.1038/s41598-019-53219-w)
Supplement: Supplementary file 1 — Supplementary Material [file 41598_2019_53219_MOESM1_ESM.pdf]

Numerical modelling of the effects of cold atmospheric plasma on mitochondrial redox homeostasis and energy metabolism

Tomoyuki Murakami*

[1]Seikei University, Department of Systems Design Engineering, Faculty of Science and Technology, 3-3-1 Kichijoji-Kitamachi, Musashino, Tokyo 180–8633, Japan
*tomo-murakami@st.seikei.ac.jp

.

Index	Reaction	Rate v_i	Constant k_i	Ref.
	Pyruvic acid oxidation			
R01	$\rightarrow$ Pyr	k_{01}	38×10^{-6} [a]	35
R02	$Pyr + NAD^+ \rightarrow AcCoA + NADH$	$k_{02}[Pyr][NAD^+]$	152 [b]	35
	TCA cycle			
R03	$OAA + AcCoA \rightarrow Cit$	$k_{03}[OAA][AcCoA]$	57142 [b]	35
R04	$Cit + NAD^+ \rightarrow KG + NADH$	$k_{04}[Cit][NAD^+]$	53 [b]	35
R05	$KG + 2NAD^+ + ADP \rightarrow OAA + 2NADH + ATP$	$k_{05}[KG][NAD^+][ADP]$	82361 [c]	35
R06	$Pyr + ATP \rightarrow OAA + ADP$	$k_{06}[Pyr][ATP]$	40 [b]	35
R07	$OAA \leftrightarrow KG$	$k_{07}([OAA] - [KG]/k_{eq})$	3.2×10^{-3} [d]	35
R08	$OAA \rightarrow$	$k_{08}[OAA]$	3.6 [d]	35
	Membrane reaction			
R09	$ATP \rightarrow ADP$	$k_{09}[ATP]$	0.005–0.1 [d]	35
R10	$H^+_{ex} \rightarrow H^+$	$k_{10}\Delta\psi$	0.426×10^3 [e]	35
R11	$ADP\ (+3H^+_{ex}) + Pi \rightarrow ATP\ (+3H^+) + H_2O$	$f([ATP],[Pi],\Delta\psi)$	–	35
R12	$NADH + \frac{1}{2}O_2\ (+10H^+) \rightarrow NAD^+ + H_2O\ (+10H^+_{ex})$	$f([NADH],\Delta\psi)$	–	35
	ROS regulation			
R13	$NADH + O_2\ (+H^+) \rightarrow NAD^+ + H_2O_2$	$k_{13}[NADH][O_2]$	3.0 [a]	33
R14	$H_2O_2 + Fe^{3+} \rightarrow coI$	$k_{14}[H_2O_2][Fe^{3+}]$	1.8×10^7 [a]	33
R15	$coI + ARH \rightarrow coII + AR^*$	$k_{15}[coI][ARH]$	1.5×10^5 [a]	33
R16	$coII + ARH \rightarrow Fe^{3+} + AR^*$	$k_{16}[coII][ARH]$	5.2×10^3 [a]	33
R17	$NAD^* + O_2 \rightarrow NAD^+ + O_2^-$	$k_{17}[NAD^*][O_2]$	2.0×10^7 [a]	33
R18	$O_2^- + Fe^{3+} \rightarrow coIII$	$k_{18}[O_2^-][O_2]$	1.7×10^7 [a]	33
R19	$2O_2^-\ (+2H^+) \xrightarrow{(SOD)} H_2O_2 + O_2$	$k_{19}[O_2^-]^2$	2.0×10^7 [a]	33
R20	$coIII + NAD^* \rightarrow coI + NAD^+$	$k_{20}[coIII][NAD^*]$	4.0×10^7 [a]	33
R21	$2NAD^* \rightarrow NAD_2$	$k_{21}[NAD^*]^2$	6.0×10^7 [a]	33
R22	$Fe^{3+} + NAD^* \rightarrow Fe^{2+} + NAD^+$	$k_{22}[Fe^{3+}][NAD^*]$	1.8×10^6 [a]	33
R23	$Fe^{2+} + O_2 \rightarrow coIII$	$k_{23}[Fe^{2+}][O_2]$	1.0×10^5 [a]	33
R24	$AR^* + NADH \rightarrow ARH + NAD^*$	$k_{24}[AR^*][NADH]$	7.0×10^5 [a]	33
R25	$O_{2,eq} \rightarrow O_2$	$k_{25}[O_{2,eq}]$	6.0×10^{-3} [d]	33
R26	$O_2 \rightarrow O_{2,eq}$	$k_{26}[O_2]$	6.0×10^{-3} [d]	33

Table S1. Biochemical reactions involved in the present simulation. Units. a: $M\cdot s^{-1}(= mol\cdot L^{-1}\cdot s^{-1})$, b: $M^{-1}\cdot s^{-1}$, c: $M^{-2}\cdot s^{-1}$, d: s^{-1}, e: $M\cdot s^{-1}\cdot V^{-1}$.

Reactant	Abbreviation	Initial concentration	Ref.
Phosphate	Pi	2.44 [a]	35
Pyruvate	Pyr	0.14 [a]	35
Citrate	Cit	0.40 [a]	35
Acetyl-CoA	AcCoA	0.07 [a]	35
α-ketoglutarate	KG	0.25 [a]	35
Oxaloacetate	OAA	0.50×10^{-2} [a]	35
Adenosine triphosphate	ATP	3.23 [a]	35
Adenosine diphosphate	ADP	0.93 [a]	35
Reduced nicotinamide adenine dinucleotide	NADH	0.13 [a]	35
Oxidised nicotinamide adenine dinucleotide	NAD^+	0.94 [a]	35
Nicotinamide adenine dinucleotide radical	NAD^*	0.9×10^{-3} [b]	33
Nicotinamide adenine dinucleotide dimer	NAD_2	1.07 [b]	33
Ferrous peroxidase iron(II)	Fe^{2+}	1.20×10^{-2} [b]	33
Ferric peroxidase iron(III)	Fe^{3+}	0.60 [b]	33
Enzyme intermediates compound I	coI	7.10×10^{-4} [b]	33
Enzyme intermediates compound II	coII	0.02 [b]	33
Enzyme intermediates compound III	coIII	0.77 [b]	33
Aromatic compound	ARH	2.99×10^{2} [b]	33
Aromatic compound free radical	AR^*	1.11×10^{-3} [b]	33
Oxygen molecule	O_2	6.85 [b]	33
Superoxide anion	O_2^-	8.60×10^{-4} [b]	33
Hydrogen peroxide	H_2O_2	0.90×10^{-2} [b]	33

Table S2. Initial concentrations. Units. a: mM, b: μM. ATP+ADP=4.16 mM. NADH+NAD^+=1.07 mM.
